# Pattern Recognition in Neural Networks with Competing Dynamics: Coexistence of Fixed-Point and Cyclic Attractors

**DOI:** 10.1371/journal.pone.0042348

**Published:** 2012-08-10

**Authors:** José L. Herrera-Aguilar, Hernán Larralde, Maximino Aldana

**Affiliations:** 1 Instituto de Ciencias Físicas, Universdad Nacional Autónoma de México, Cuernavaca, Morelos, México; 2 Facultad de Ciencias, Universidad Autónoma del Estado de Morelos, Cuernavaca, Morelos, México; 3 FAS Center for Systems Biology and The David Rockefeller Center for Latin American Studies, Harvard University, Cambridge, Massachusetts, United States of America; National Research & Technology Council, Argentina

## Abstract

We study the properties of the dynamical phase transition occurring in neural network models in which a competition between associative memory and sequential pattern recognition exists. This competition occurs through a weighted mixture of the symmetric and asymmetric parts of the synaptic matrix. Through a generating functional formalism, we determine the structure of the parameter space at non-zero temperature and near saturation (i.e., when the number of stored patterns scales with the size of the network), identifying the regions of high and weak pattern correlations, the spin-glass solutions, and the order-disorder transition between these regions. This analysis reveals that, when associative memory is dominant, smooth transitions appear between high correlated regions and spurious states. In contrast when sequential pattern recognition is stronger than associative memory, the transitions are always discontinuous. Additionally, when the symmetric and asymmetric parts of the synaptic matrix are defined in terms of the same set of patterns, there is a discontinuous transition between associative memory and sequential pattern recognition. In contrast, when the symmetric and asymmetric parts of the synaptic matrix are defined in terms of independent sets of patterns, the network is able to perform both associative memory and sequential pattern recognition for a wide range of parameter values.

## Introduction

Neural networks were originally developed to model the behavior of the brain. However, due to the great complexity of the brain's neural circuitry and of the synaptic interactions, it was necessary to propose simplified models, such as the McCulloch-Pitts model [1] and the Hopfield model [2] which, although simple, still capture some important characteristics of the neuronal dynamics. One important question in this field is how and when a neural network is able to memorize a given set of patterns. Two main different mechanisms to store information in a neural network have been identified, to wit, the Associative Memory (AM) on the one hand, and the Sequential Pattern Recognition (SPR) on the other hand. The neural network performs AM when its dynamical attractors are fixed points, each corresponding to one of the patterns that we want to store in the network. This type of dynamic behavior is characterized by a symmetric interaction matrix that contains the connection strength between the neurons. Some examples of this dynamics are the Hopfield model and the Little model [2],[3], [4], [5]. Contrary to the above, in SPR the network memorizes a fixed set of patterns which are retrieved in certain order in time. From a dynamical point of view, this corresponds to a cyclic attractor consisting of the sequence of patterns stored in the network in a given order. A necessary condition for SPR to occur is that the matrix of neuron-neuron interactions has to be asymmetric. A well known example of this type of dynamics is the asymmetric Hopfield model [2], [6].

Several dynamical phases have been identified in both the AM and SPR models. Broadly speaking, these phases characterize how well the network can recognize its set of patterns, and the type of memory, i.e., whether AM or SPR. Both AM and SPR have been widely studied separately. Nonetheless, there is evidence showing that in real neural networks the synaptic connections are neither fully symmetric nor fully asymmetric [7], [8]. Rather, they can be considered as a mixture of these two cases, generating an interaction network with a complex topology. Additionally, there is evidence that the brain is capable to perform both AM and SPR [8], [9]. For instance, recalling the color of a simple object would be an example of AM, whereas recalling the digits in a phone number in the proper order would constitute an example of SPR. Since these two types of pattern retreival coexist in the brain, several authors have introduced modifications to the Hopfield model in order to obtain both types of pattern retrieval within the same network [10], [11], [12]. One approach to this problem was proposed by Coolen and Sherrington in Ref. [10]: They introduced a model in which the interaction matrix has two parts, one symmetric and one asymmetric. These two parts are weighted by a *mixture parameter* 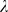, in such a way that the interaction matrix 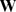, (also called the synaptic matrix), can be written as
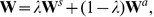
(1)where 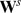 and 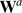 are symmetric and asymmetric matrices, respectively. For 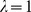 only the symmetric part is present (the classical Hopfield Model) and therefore the network performs AM, while for 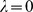 only the asymmetric part survives (the asymmetric Hopfield Model) and the network performs SPR. For intermediate values of 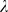, there is a competition between the symmetric and asymmetric parts of the synaptic matrix. One of the main questions in this model, which we will refer to as the *Coolen-Sherrington model*, (or the CS model for short), is how the network dynamics transit from AM to SPR as 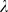 varies from 1 to 0. The point is that for 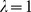 all the patterns are stored as independent attracting fixed points, whereas for 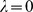 the patterns are stored as part of a single large cyclic attractor. Is this transition from AM to SPR continuous or discontinuous? Can some of the patterns be stored in a cyclic attractor whereas some other patterns are stored as fixed point attractors? As we will see, the answer to these questions depends on the definition of the symmetric and asymmetric parts of the synaptic matrix.

In the original CS model the symmetric part 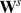 and the asymmetric part 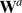 are correlated because they are defined in terms of the same set of patterns (see the definition of the model in the next section). Additionally, Coolen and Sherrington studied this model for the particular case where the number of patterns stored in the network is smaller that the number of neurons (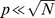). In terms of the load parameter 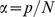, the above condition corresponds to 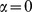 in the thermodynamic limit 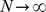 (this regime is termed *non saturated*.) Within this regime, Coolen and Sherrington found that for parallel updating and for 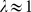, the network dynamics exhibit only fixed point attractors, i.e. the network performs AM. However, when 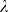 is decreased, a first order phase transition appears: Below a certain critical value 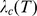 that depends on the temperature 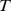, the dynamical trajectories end up either in cyclic attractors (the networks exhibits SPR), or in stable mixed states that consist of combinations of the desired patterns. However, no coexistence of AM and SPR was found.

Afterwards, in Ref. [13] the authors studied the CS model using correlated patterns. They found that it is possible to have SPR (the stable cycle limit is still present) when the correlation between the patterns is small. In the case of AM the network goes to a fixed point attractor but this attractor does not coincide with any of the desired patterns. Metz and Theumann [14], [15] presented a full study of the stability of the patterns in a multi-layered neural network with competition between AM and SPR, finding the phase space regions where the network performs AM, SPR and the region for the spin-glass solutions (SGS), but no coexistence between AM and SPR was found either. By “coexistence” of AM and SPR we mean that some of the patters are stored as fixed point attractors while other patterns are stored in larger cyclic attractors. When such a coexistence does not exist, then all patterns are stored either as independent fixed point attractors or as a single large cyclic attractor. Recently, the same authors in [16] studied a model similar to the CS model by means of the generating functional technique. They present a study of the stationary states and the different regions on the phase space where either fixed points or cyclic attractors are attained.

It is important to note that the lack of coexistence of AM and SPR in the original CS model is not a trivial result. The synaptic weights are a weighted mixture of symmetric and asymmetric matrices. Therefore, especially for intermediate values of the mixture parameter 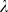, it could have happened that some of the patterns were recognized as fixed-point attractors whereas *some other* patterns were recognized in sequential order. But this was not the case: either all the patterns are fixed point attractors or all of them form a *huge* cyclic attractor (remember that we are working in the saturated regime where the number of stored patters is a finite fraction of the number of neurons: 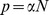, which becomes infinite in the thermodynamic limit 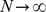). This all-or-none behavior was not expected for the original CS model and deserved a careful study carried out by several authors.

Using the generating functional formalism developed in Refs. [17], [18], [19], [20]–[21] we investigate how the network transits from pure AM to pure SPR for the CS model, first in the case in which the symmetric and asymmetric parts of the synaptic matrix are correlated (defined in terms of the same set of patterns) and then when they are independent (defined each in terms of different sets of patterns). Both cases are studied for systems in which the number of patterns 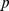 is a finite fraction of the total number of neurons 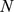, namely, in the *saturated regime* for which 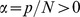 even in the thermodynamic limit 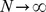. We compute the phase space over the parameters 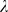, 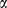 and the temperature 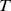, finding the regions where the networks performs AM and/or SPR, as well as the spin-glass region, for different values of the mixing parameter 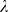. As might have been expected, we find that when 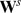 and 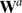 are correlated, the network either performs AM or SPR, but it is incapable to perform both for the same set of parameter values. In contrast, when 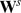 and 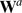 are independent of each other, AM and SPR coexist within a large region of the parameter space. We present a complete explicit characterization of the different phases, as well as the transition between AM and SPR when these behaviors coexist.

In the next section we present the two versions of the CS model we study in this work. In Sec. we compute the dynamical equations that determine the temporal evolution of the network using the generating functional formalism developed in Refs. [17], [18], [19], [20]. In Sec. we present the results for the original CS model and determine the structure of the parameter space identifying the regions of highly correlated, weakly correlated and spin-glass solutions. We do this for the AM and SPR dynamics and show that these two types of pattern retrieval do not coexist. In Sec. we present analogous results but for the modified version of the CS model, and we show that in this case AM and SPR dynamics do coexist. Finally, in Sec. we summarize our results.

## Materials and Methods

### Model Definition

The network under consideration consists of 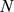 binary neurons, 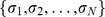, each acquiring the values 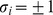. We will denote as 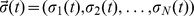 the dynamical state of the entire network at time 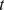. The interaction between the neurons is determined by the function
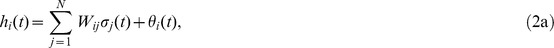
where the 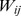, the components of the synaptic matrix 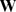, are defined in terms of the patterns that we want to store in the network. The variables 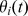 represent external fields. We will come to the precise definition of the synaptic weights 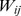 in a moment. For the time being, let us assume that these synaptic weights have already been defined. Then, the network dynamics are given by the synchronous updating of all the network elements in such a way that
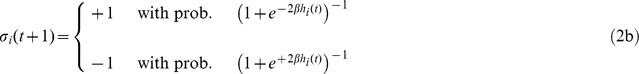
where 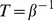 is the statistical “temperature”. The above updating rule is equivalent to saying that the *transition probability* 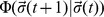 for the entire network to change from the state 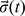 at time 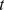 to the state 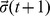 at time 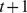 is
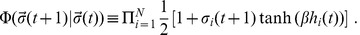
(2c)


In order to define the synaptic weights 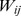, we will assume that they consist of a symmetric part 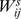 and an asymmetric part 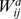 as
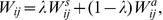
(3)where 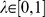 is the *mixture parameter*. In the original CS model, each of these parts are defined in terms of a unique set of 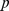 uncorrelated patterns, 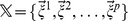, as
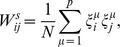
(4a)

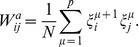
(4b)where each pattern 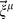 is a distinct vector of 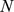 binary digits; 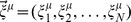, and each entry can take the values . For the purpose of this work, these binary sequences are generated randomly. In the last expression, the sum over the patterns is modulo 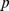, i.e. 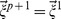.

In order to make the two types of pattern retrieval AM and SPR coexist in the same network, in this work we introduce a variation of the CS model by using *two different* sets of uncorrelated patterns, 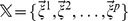 and 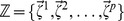. Each pattern is uncorrelated with all the other patterns in its own set and also with all the patterns in the other set. We can use these two independent sets of patterns to define 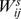 and 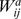 independently of each other as
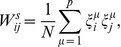
(5a)

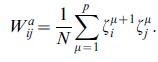
(5b)


Use of independent patterns in Eq. (5) has been studied previously for the special case of 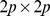 matrices [21], [20]. Here, we extend these calculations for general matrices and compute the whole phase space.

The quantity that determines how close the state 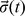 is from a given pattern, say 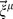, is the overlap function 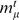, defined as
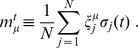
(6)


Thus, if 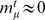, then 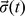 and 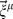 are very different and uncorrelated, whereas if 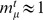 then 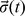 and 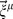 are almost identical. Finally, if 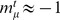 then 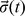 and 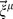 are specular copies of each other (i.e. they are fully anticorrelated).

In what follows, we analyze the models given in Eqs. (2)–(5) both numerically and by the generating functional approach, from which we derive the equations that rule the dynamical evolution of each system and determine the structure of their respective phase spaces.

## Results

### Numerical simulations

**Figure 1:**
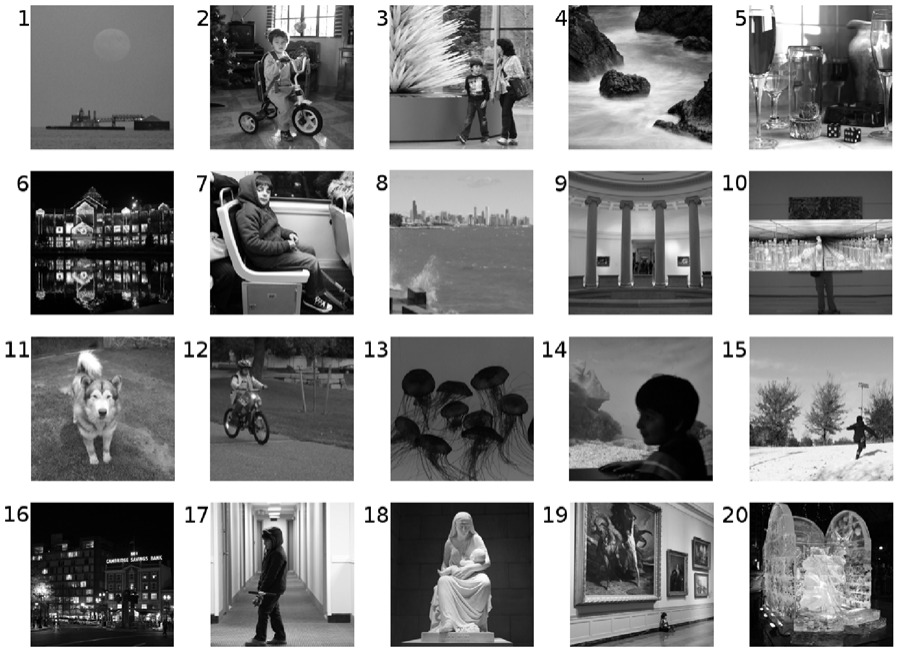
Twenty patterns used in the numerical experiments. Each pattern is a digitalized image with 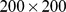 pixels, each with 8 bits of depth. The binary strings directly reproducing these images were stored in the synaptic matrix 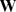 without any previous randomization. In the case when 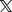 and 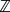 are two independent sets, patters 1 to 10 were used for 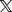 and patterns 11 to 20 for 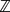. When 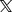 and 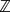 are the same set, patterns 1 to 10 were used for both.

Before introducing the mathematical formalism to analyze the different models presented in the previous section, we illustrate here the coexistence of the two types of dynamics, SPR and AM, with a numerical simulation. For this, we constructed a neural network that can store 10 patterns 
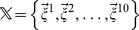
 as independent fixed points (for the AM recognition), and 10 patterns 
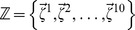
 in a single cyclic attractor (for the SPR). Each pattern is a grayscale digitalized image 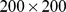 pixels in size, and each pixel has a depth of 8 bits in order to encode 256 shades of gray needed for the black and white image. Fig. 1 shows the 20 patterns used in our numerical simulations. The neural network thus consists of 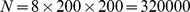 binary variables. Furthermore, although in theory one assumes that each neuron is connected to each other neuron in the network, in our case this would give a very large connectivity matrix with 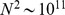 independent entries. Such a large synaptic matrix is not necessary to store 20 patterns. Hence, in our simulations we worked with networks where each neuron receives inputs only from 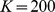 other neurons randomly chosen with uniform probability from the entire network. Once the 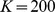 input connections of each neuron have been randomly assigned, they do not change throughout the dynamics of the network. Thus, the synaptic matrix 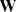 is a sparse matrix constructed according to Eq. (1), where 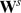 and 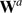 are given as in Eq. (4), or as in Eq. (5), depending on whether 
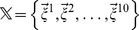
 and 
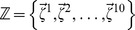
 are the same set of patterns, or two different independent sets, respectively.

**Figure 2:**
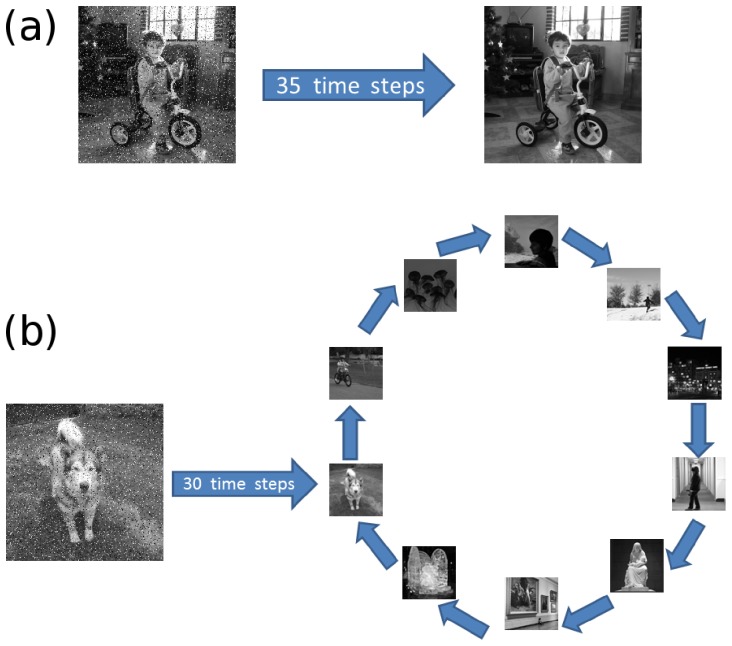
Schematic representation of the AM and SPR dynamics in the numerical simulations. (a) For the AM dynamics, we start the network with a state that differs 10% from one of the stored patterns 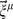 in 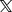 (noisy image on the left). Then, we evolve the network 35 time steps and compute the overlap 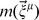 between the final network state and the pattern 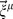. We do this for each of the 10 patterns in 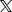 and compute the average overlap over these 10 patterns. (b) For the SPR dynamics we proceed in a similar way, starting the network from a state that differs 10% from one of the stored patterns 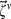 in 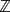. We let the system evolve for a transient time of 30 time steps (three periods of the supposedly cyclic attractor). After that, we run the dynamics for 10 time steps (one period of the supposed cycle) and, as the network traverses the cycle, compute the overlaps 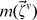 of the network state with each one of the 10 patterns in 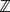 (retrieved in the order from 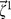 to 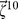). The final overlap in this case is the average of the overlaps throughout the cycle.

For the AM dynamics, we initialize the network in a state that differs 10% from a given pattern 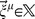, as illustrated in Fig. 2a. Then we run the dynamics for a transient time of 35 time steps, after which we compute the overlap 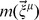 between the network state and the pattern 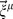. We do this for every pattern in the set 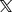 and compute the average overlap 
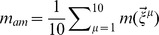
. For the SPR dynamics we proceed in a similar way (see Fig. 2b), starting the network in a state that differs 10% from a given pattern 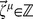 and then running the dynamics for a transient time of 30 time steps. Then, we run the dynamics for another 10 time steps, which would be the length of the cycle formed by the patterns 
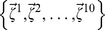
, and sequentially compute the overlap 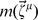 between the network state and each of the corresponding patterns in the set 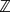, assuming that these patterns are retrieved in the order from 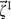 to 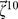. The overlap is again the average 
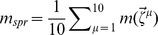
.

**Figure 3:**
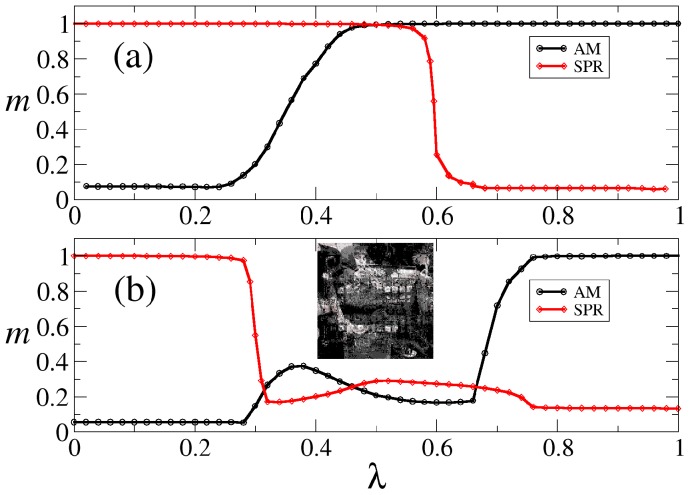
Pattern recognition measured by the overlap. This figure shows the graph of the overlap for the AM dynamics (black curve) and SPR dynamics (red curve) as a function of the mixture parameter 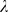 in two cases: (a) when the two sets 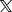 and 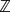 consist of different and independent patterns, and (b) when 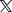 and 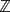 consist of exactly the same set of 10 patterns. It is clear that in (a) the network can perform both AM and SPR dynamics almost perfectly within the interval 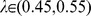. However, in (b) there is no value of 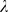 for which the network can perform both AM and SPR. By contrast, in the intermediate region 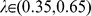 the network always invariably falls into a “frustrated” state consisting of a random superposition of the patterns stored in the network, as the one shown in the inset.

Fig. 3a shows the results of the simulation for the case when 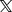 and 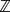 are different sets of patterns (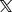 consists of the patterns 1 to 10 whereas 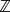 contains the patterns 11 to 20 of Fig.1). It is clear for this case that in the interval 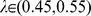 the network can perform both associative memory and sequential pattern recognition almost perfectly (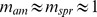). Depending on the initial condition, the network will retrieve one of the fixed point patterns 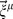 in 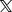, or it will retrieve all the patterns 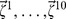 in 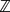 in a cyclic order. Thus in this case AM and SPR coexist for 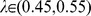. By contrast, Fig. 3b shows that when the sets 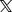 and 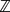 contain the same set of patterns (patterns 1 to 10 of Fig. 1), namely, when 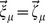 for 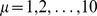, then there is no value of 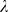 for which 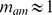 and 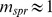 at the same time. Therefore, in this case the network cannot perform AM and SPR and these two types of dynamics do not coexist.

Finally, it is worth mentioning that the relatively high values of the overlap observed in the interval 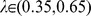 in Fig. 3b are due to the fact that in this region the network invariably falls into a “frustrated” state consisting of a random superposition of different patterns, as the one shown in the inset. Therefore, in this region the network always has a non negligible overlap with any of the stored patterns, producing a relatively high value of the overlap.

### Generating functional approach

We use the standard generating functional approach [17], [18], [19], [20], [21] to derive the dynamical mapping that determines the temporal evolution of the average of the overlap 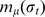. This formalism allows us to derive state equations for the macroscopic variables of the system at finite temperature 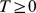. For the sake of completeness, we outline the procedure following [17]. Thus, we define the generating functional 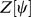 as:
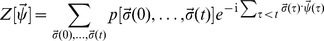
where 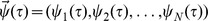 is a set of auxiliary variables used to derive the macroscopic parameters that characterize the system, and 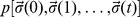 denotes the probability of taking the path with initial condition 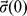 and final condition 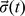. (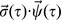 denotes the usual dot product.)

Since Eq. (2c) defines a Markov process, the probabilities 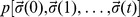 can be expressed as a product of the transition probabilities 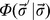, which leads to


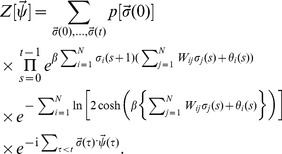
To uncouple the terms 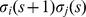, auxiliary variables 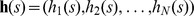 are introduced, representing the local fields 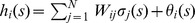 at each neuron at each time step. In terms of these, the generating functional acquires the form ([17]):
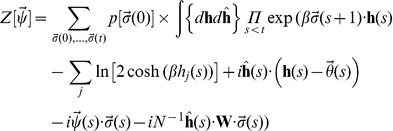
(7)where the conjugate fields 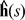 arise from the integral representation of the delta functions that enforce the values of the 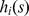.

To continue we must assume that one stored pattern is condensed at each time-step, that is, only one pattern can be highly correlated with the network. Thus, the overlap between this condensed pattern and the network state should be of order 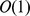, whereas for the non-condensed patterns, which play the role of quenched disorder, the overlap must be of order 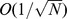. In the thermodynamic limit 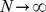 one can use Coolen's mean-field approach to average Eq. (7) over the non-condensed patterns.

Eq. (7) contains all the dynamical properties of the system. In particular, one can obtain all the relevant average quantities from the generating functional by differentiation (see Ref. [17]):
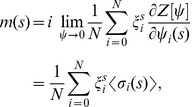
(8a)

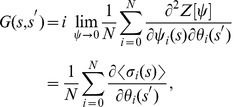
(8b)

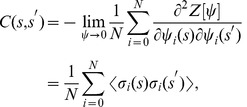
(8c)where 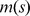 is the overlap, 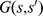 is the response function and 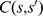 the correlation function.

### Analytic solutions for one set of patterns

As reference, we start by considering the case given in Eqs. (4), which corresponds to the original CS model where the symmetric and asymmetric parts of the synaptic matrix are correlated due to the fact that the same set of patterns 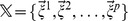 is used to define these two parts. Using the generating functional formalism, we determined the dynamical equations of the system and found the conditions for the existence of fixed points (associative memory) and limit cycles (sequential pattern recognition) for different values of the load parameter 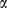, the mixing parameter 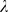, and the “temperature” 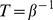. The detailed computations are shown at the Appendix S1.

#### Associative memory solutions

In order to find the regions of the parameter space where the patterns 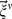 (and the corresponding anti-patterns) are fixed point attractors of the network, we look for solutions in which the final state of the network is strongly correlated with one of the patterns, say 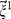. Following the procedure presented in Ref. [10], leads to the following equations for the observables:
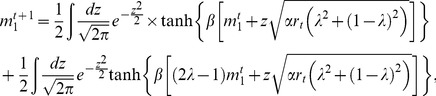
(9a)

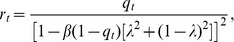
(9b)

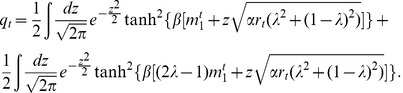
(9c)


In the above equations, 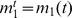 is the overlap with the first pattern, 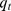 the variance of the overlap, and 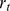 the correlation between two network states.

#### AM spin-glass solutions

**Figure 4:**
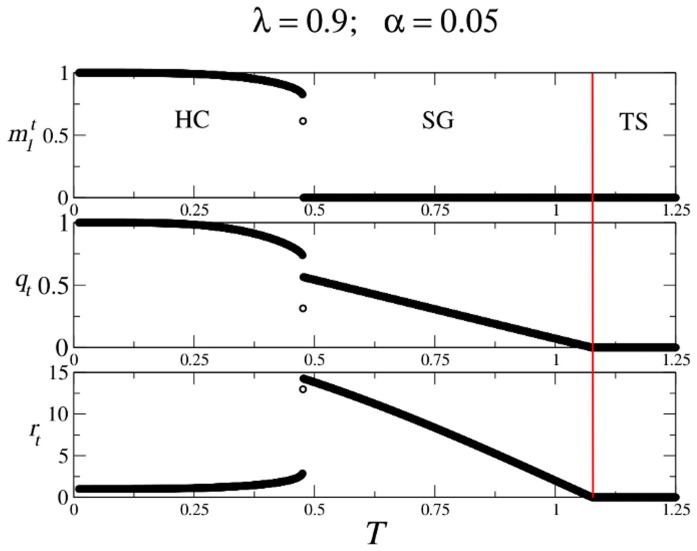
Phase transitions in the model with 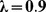
 **and** 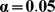 **.** Note the existence of two phase transitions, one discontinuous (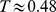) and the other continuous (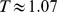). The discontinuous transition characterizes the transit from highly correlated patterns (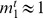) to uncorrelated ones (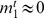) as the temperature 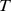 increases. The vertical line indicates the continuous transition from the spin-glass solutions to another region where only the trivial solution exists. Regions HC, SG and TS defined in the text are also indicated. The curves were obtained by solving numerically Eqs. (9).

We start the analysis of Eqs. (9) by computing the spin-glass solutions, which are characterized by 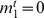, 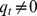, and 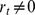. These are easily obtained at zero temperature (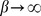), and then one can investigate the existence of spin-glass solutions at non-zero temperature by a series-expansion technique around the zero temperature solution. Since 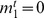, we can disregard Eq. (9a) and focus our attention only on the other equations (putting 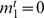). For non-zero temperature (finite 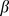), it is known that in the Hopfield model, which is obtained here for 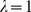, the spin-glass solutions disappear continuously as 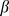 decreases via a second order phase transition. A similar behaviour is observed for 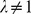, as is shown in Fig. 4, where a vertical line indicates the continuous transition from the spin-glass region SG to another region TS where only the trivial solution 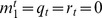 exists. Note that close to this transition 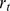 and 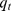 are very small. Therefore, in order to find the critical temperature at which this transition occurs, we can expand Eqs. (9c) and (9b) up to the first order in 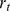 and 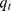, keeping 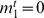. Solving the resulting equation for 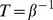 in terms of 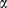 and 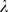, we obtain
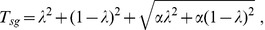
(10)where we have used 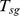 to denote that this value corresponds to the spin-glass transition temperature. The above equation is well defined for every 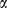 and 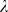 values and therefore, there is always a critical temperature at which the spin-glass phase appears.

#### AM regions

**Figure 5:**
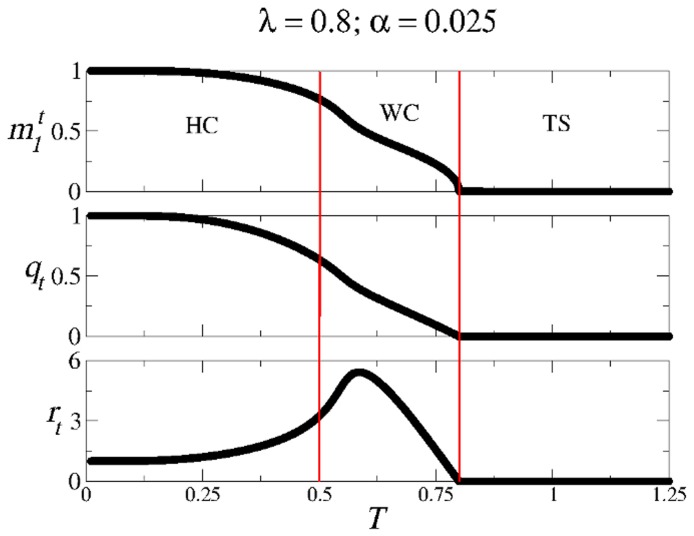
Phase transitions in the model with 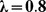
 **and** 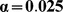 **.** In this case the three parameters 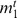, 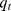 and 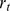 change continuously in the whole range of temperatures. As in Fig. 4, there is a continuous transition around 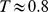 from the non-zero spin-glass solutions to only trivial solutions. However, there is another transition around 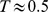 from highly correlated patterns with 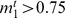, to weakly correlated ones for which 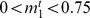. The two vertical lines enclose region WC of weak correlations defined in the text. The curves were obtained by solving numerically Eqs. (9).

The regions in which associative memory exists (AM-regions hereafter) are characterized by 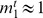. Note that the transition between the AM-region and the spin-glass region (SP-region hereafter) must be discontinuous, since in the latter 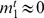. This occurs for some values of 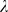 and 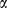, as is apparent from Fig. 4. However, for other values of the parameters the spin-glass region disappears and 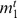, 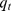, 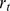 all vanish continuously, as shown in Fig. 5. It is still possible to define a region of high correlation (region HC) and another region of weak correlation (region WC) by defining thresholds to the values of the overlap 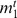. We choose the highly correlated patterns as those for which 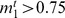, whereas for the weakly correlated patterns 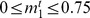.

**Figure 6:**
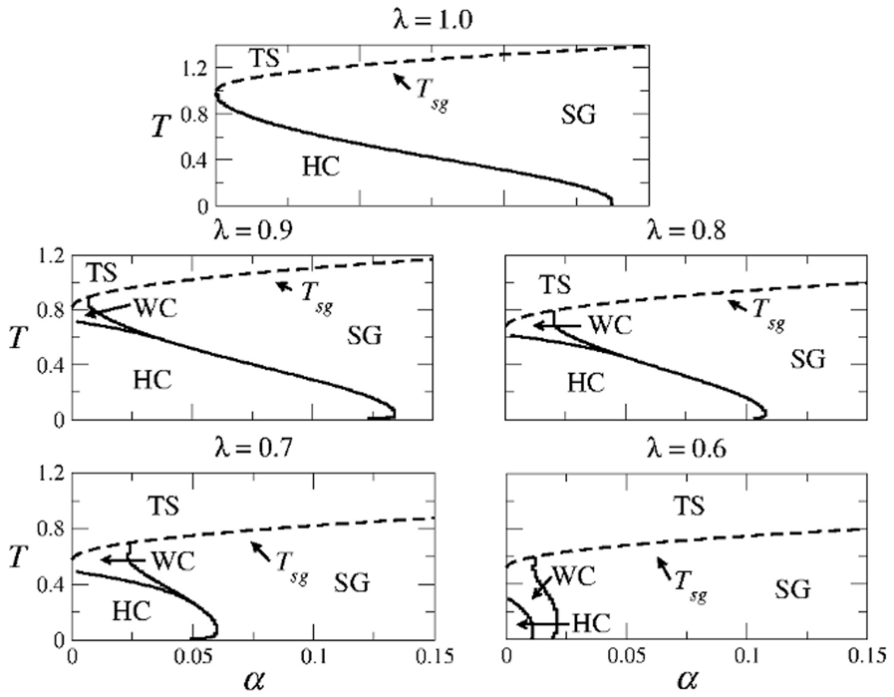
Structure of the phase space for different values of 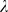
 **and for the AM dynamics.** The top panel corresponds to the well known Hopfield model [22], [23], [24]. Note that as 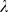 decreases, region HC of highly correlated patterns decreases, whereas region WC of weakly correlated patterns increases.

The whole structure of the parameter space for the AM dynamics, shown in Fig. 6 for different values of 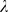, can be found by numerically solving the system of Eqs. (9). Four regions are found, which will be referred to as regions HC (high correlations), WC (weak correlations), SG (spin glass), and TS (trivial solution). Region HC is characterized by the existence of non-trivial highly correlated solutions (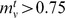). Although in this region spin-glass solutions might also exist, the network performs AM since the highly correlated solutions are always preferred. In region SG the network is not capable to perform AM. Rather, it always falls into spin-glass solutions. In region TS only the trivial solution exists. Finally, region WC is a transition region where the highly correlated patterns continuously become weakly correlated. Even when there are non-trivial solutions in region WC, it is not possible to have AM because the final pattern has at most 75% of its neurons correct with respect to the desired final state. It is worth emphasizing that in the transition from region HC to SG the overlap 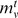 varies discontinuously from 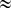 1 to 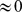, see Fig. 4, whereas in the transition from region HC to WC to TS the overlap varies continuously from 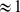 to 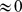, see Fig. 5).

### Sequential pattern recognition solutions

Now we find the regions of the parameter space where sequential pattern recognition exists. In these regions, the patterns 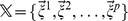, form a cyclic attractor with period 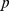, i.e. 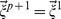. We will look for solutions of Eq. (7) in which the state of the network at time 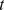 is strongly correlated only with 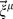, at time 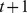 it is strongly correlated only with 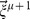, and so on until the time step 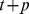, at which the state of the network is strongly correlated only with 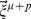, and the cycle starts over again. The condition that at each time the network state is correlated with only one pattern can be written as
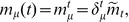
(11)where 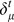 is the Kronecker delta function and 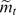 is a quantity such that 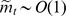. The calculation again follows along the lines presented in Ref. [10]. The final set of equations for 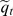, 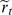, and 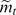 is
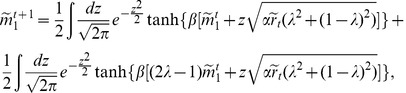
(12a)

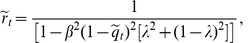
(12b)

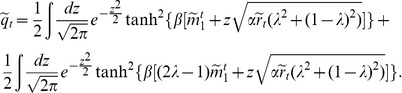
(12c)


The system of equations above is always satisfied by the trivial solution 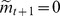, 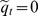 and 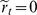. However, depending on the values of the parameters 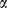, 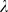 and 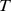, the trivial solution can be stable or unstable. We are of course interested in the regions of the parameter space where the set of patterns 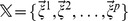 is retrieved in the specified order. This corresponds to solutions for which 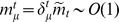 with no restrictions on 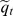 and 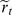. The *spin-glass* solutions are also important in the phase diagram. They are obtained from the system of Eqs. (12) by imposing the conditions 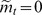, 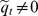. Before presenting the different regions of the phase space, in the next section we focus on the calculation of the critical value of the capacity 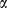 in the deterministic case of zero temperature (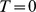, 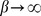), and for purely asymmetric synaptic weights, i.e. 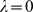.

#### SPR dynamics at zero temperature

For 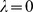 the system of Eqs. (12) becomes
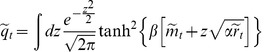
(13a)

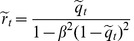
(13b)

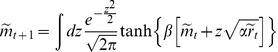
(13c)


The last three equations coincide with the ones found in Ref. [6]. Let us define 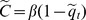. Note from Eq. (13a) that in the limit 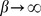, the 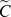 remains finite and is different from zero. Thus, using the saddle point approximation in the limit 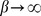, the system of equations (13) reduces to
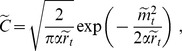


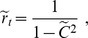


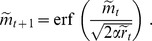



By defining 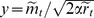, the above equations can be solved for 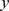, which gives
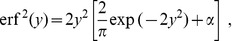
(14)which is equivalent to
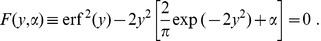
(15)


**Figure 7:**
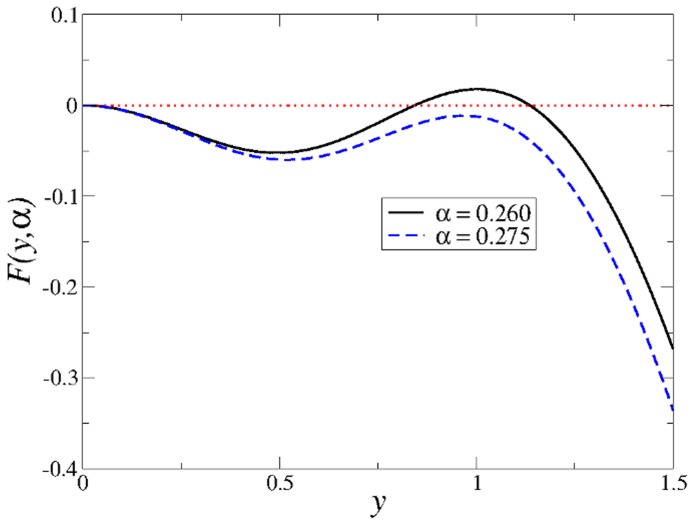
Family of curves 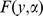
 **defined in Eq. (15).** For 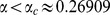 there is a non-trivial solution of 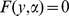 (solid line). For 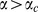 only the trivial solution exists (dashed line).

The preceding equation has non trivial solutions as long as 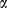 is smaller than a critical value 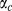 (see Fig. 7). This critical value gives the maximum storage capacity of the network. Beyond that value, SPR cannot occur. By solving numerically Eq. (15) one finds 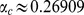, which is in agreement with the value found in Ref. [17].

#### SPR spin-glass solutions

An analysis similar to that in Sec., but now using Eqs. (12), leads to the following expression for the spin-glass transition temperature:
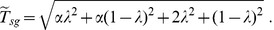
(16)


Again this transition temperature is well defined for all values of the parameters, and therefore there is always a finite temperature at which the spin-glass phase appears.

#### SPR regions

**Figure 8:**
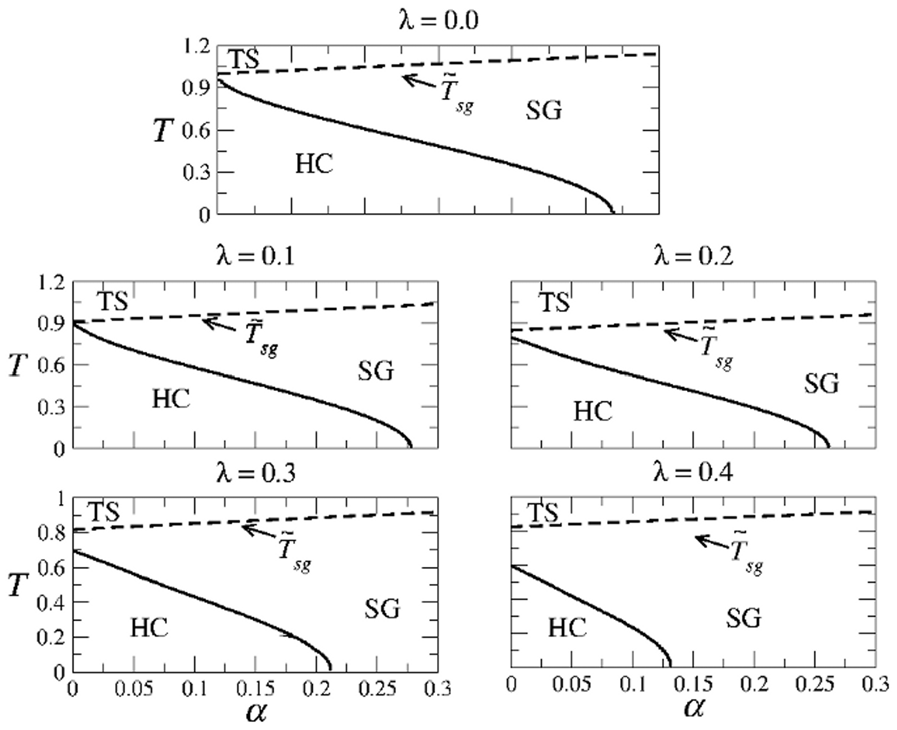
Structure of the phase space for the SPR case and different values of 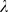
. In region HC the highly correlated solutions coexist with the spin-glass solutions. In region SG only spin-glass solutions exist, and in region TS only the trivial solution exists.

Fig. 8 shows the regions in the parameter space where different types of solutions are obtained in the SPR case. There are only three regions, which are referred to as HC, SG and TS. Region HC is where highly correlated solutions, characterized by 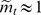, coexist with Spin-Glass solutions. In region SG the highly correlated states disappear and only the Spin-Glass solutions exist. Finally, in region TS only the trivial solutions exist. Note that in this case there is no region of weakly correlated solutions, (as for AM). Therefore, the highly correlated solutions always disappear discontinuously through a first-order transition from region HC to region SG. In contrast, the spin-glass solutions vanish continuously from region SG to region TS.

### Coexistence of AM and SPR dynamics

**Figure 9:**
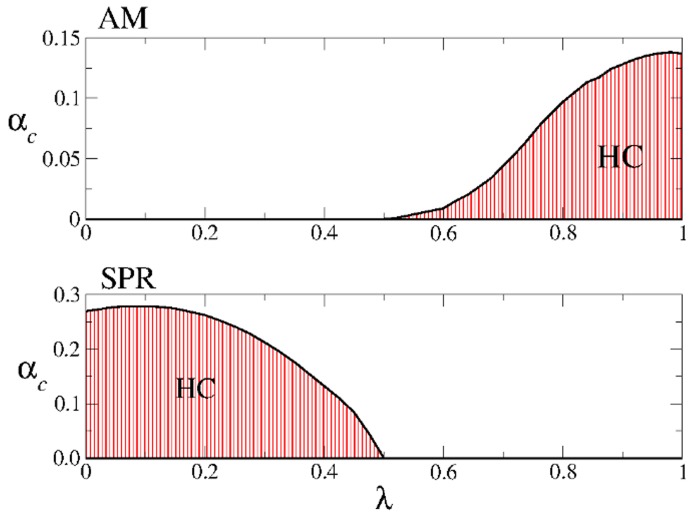
Regions in the 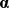
--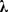 **parameter space of highly correlated solutions for the AM (top) and SPR (bottom) dynamics in the original CS model at zero temperature.** The shaded areas are the regions where the highly correlated solutions exist. The solid curves in black are the critical lines 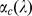 at which these solutions disappear. These curves were obtained by numerically solving Eqs. (9) and (12). Note that there is no overlap between the shaded region corresponding to AM and the one corresponding to SPR.

The analysis presented so far shows that, unsurprisingly, the original CS model is unable to perform both AM and SPR dynamics for the same value of 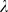. This is illustrated in Fig. 9 for the case of zero temperature, but the same happens for 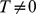. This is obviously due to the fact that the same set of patterns is used to define both the symmetric and asymmetric parts of the synaptic matrix. But the symmetric part 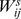 is responsible for the AM dynamics, whereas the asymmetric part 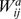 is involved in the SPR dynamics, and it is impossible for a given pattern 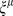 to be a one-state attractor (AM), and at the same time to belong to a cyclic attractor (SPR). Thus both dynamics cannot coexist in the original CS model. To circumvent this problem, we have modified the original CS model by defining 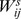 and 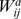 using two independent sets of patterns, as in Eq. (5).

### Analytic solutions for two independent sets of patterns

In this section we present the results of the modified CS model in which the symmetric part 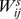 of the synaptic matrix is defined in terms of a set of patterns 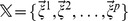, whereas the asymmetric part 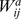 is defined using a different set of patterns 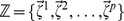, as in Eq. (5). This gives the network the possibility to perform AM and SPR dynamics independently of each other. The mathematical formalism is completely analogous to the one used in the previous section. As for the original CS model, here we present first the AM solutions and afterwards the SPR ones.

#### AM solutions

**Figure 10:**
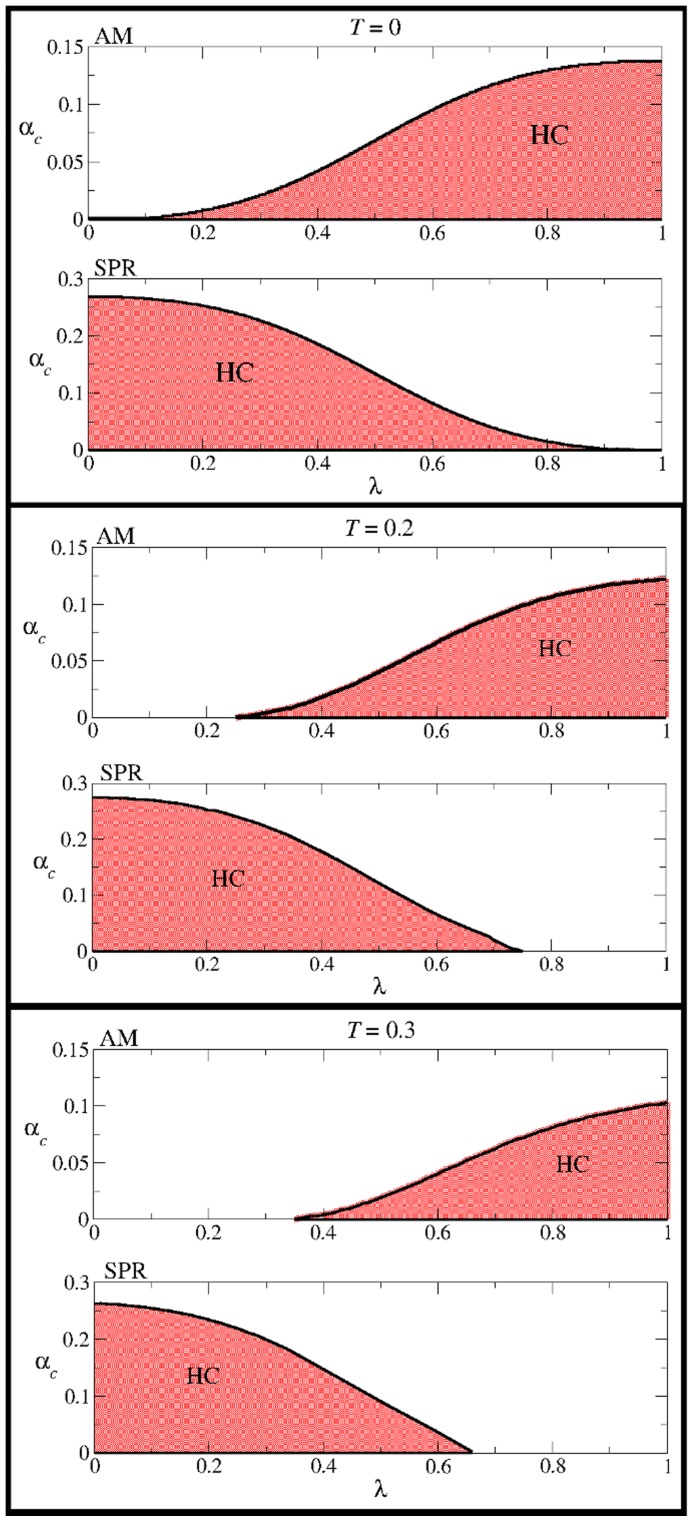
The critical value 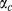
 **as a function of the mixing parameter** 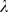 **for different temperatures.** In each panel, the upper graph corresponds to AM and the lower graph to SPR. The shaded regions corresponds to the highly correlated solutions. The solid curves for 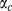 were obtained by solving numerically Eqs. (18) and (21), respectively. Note that in each case the intersection of the highly correlated regions for the AM and SPR dynamics is not empty, which indicates the coexistence of AM and SPR even at non-zero temperature.

To obtain the AM solutions we again demand that the network state is highly correlated with only one of the patterns 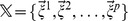, say 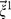. Thus, the overlap 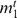 between the network state and 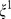 must satisfy that 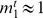, whereas all the other overlaps 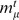 must be of order 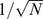. Under such circumstances, the equations that determine the stationary state of the network, equivalent to Eqs. (9), are:
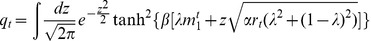
(17a)

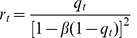
(17b)

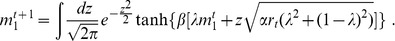
(17c)where all the variables have the same definition as in the previous section. Clearly, the above equations are simpler than the corresponding ones in the original CS model, Eqs. (9). In particular, for 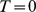 the set of equations (17) can be written as a single equation
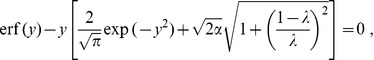
(18)where 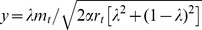. The preceding equation determines the critical value 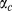 of the load parameter below which highly correlated solutions exist, namely, where the network performs AM dynamics. This critical value, which is a function of 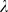, is the maximum value for which Eq. (18) has nontrivial solutions. By solving numerically the above equation, we obtain the regions depicted in Fig. 10 for several temperatures (top graphs in each panel).

From Eqs. (17) it follows that at zero temperature the spin-glass solutions always exist for any values of 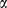 and 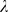. Indeed, it is easy to see that in the limit 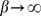, the set of Eqs. (17) has the non-trivial solution
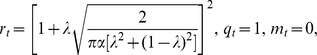
which is well defined for all values of 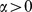 and 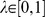. Now, to find the transition temperature 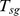 at which the spin-glass solutions continuously disappear, we expand Eqs. (17a) and (17b) in powers of 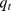 and 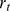, retaining only the first order terms and keeping 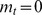, which gives
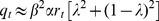


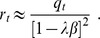



From the above equations we obtain that the spin-glass transition temperature is
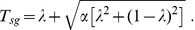
(19)


**Figure 11:**
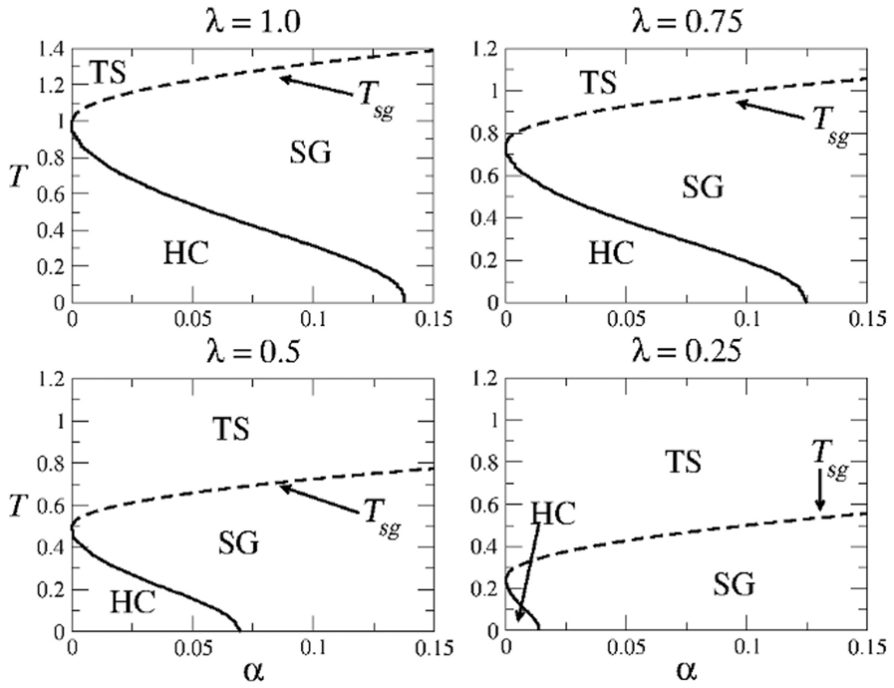
Structure of the AM phase space for different values of 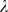
 **in the modified CS model.** The first panel for 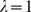 corresponds to the well known symmetric Hopfield model. Note that as 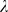 increases, region HC of highly correlated patterns decreases. However, it remains of considerable size even for 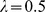, where the AM and SPR dynamics equally compete. Note also that there is no region WC of weakly correlated solution.

The previous analysis, together with numerical solutions of the system of Eqs. (17), allow us to determine the structure of the 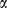-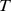 parameter space for different values of 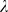. This structure in shown in Fig. 11, which reveals the existence of only three regions: Region HC is where the highly correlated solutions exist and are stable, whereas in region SG the highly correlated solutions disappear and only the spin-glass solutions exist. Finally, in region TS only the trivial solution is found. Note that, contrary to what happens in the original CS model, in this case there is not a region WC of weakly correlated solutions. Note also that as the mixing parameter 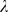 decreases, the region HC remains of considerable size. Therefore, the network can perform AM in a wide range of values of 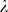.

#### SPR solutions

The SPR solutions are those for which the set of patterns 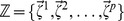 is retrieved in the specified order. This corresponds to solutions for which the overlap between the state of the network and the pattern 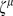 satisfies 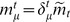, where 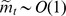. On the other hand, the overlap with the patterns 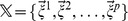 will be of order 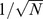. Taking these considerations into account, the equations that determine the cyclic behaviour of the network are
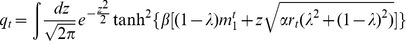
(20a)

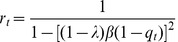
(20b)

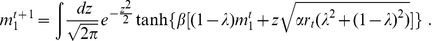
(20c)


Through a calculation similar to the one used in Sec., it can be shown that in the limit 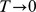 the system of Eqs. (20) reduces to
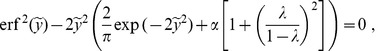
(21)where 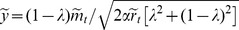. This equation has non-trivial solutions as long as 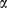 is smaller than a critical value 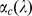 that depends on 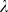. By numerically solving Eq. (21) we obtain the curve 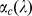 plotted in the bottom graphs in each panel of Fig. 10, in which the shaded area corresponds to the region of highly correlated solutions. Note from this figure that the shaded regions corresponding to AM and SPR do intersect over a large region of parameter values. This indicates that in the modified CS model, AM dynamics do coexist with SPR dynamics for a wide range of values of the mixture parameter 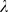.

On the other hand, the spin-glass solutions at zero temperature are given by
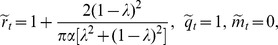
which shows that the spin-glass solutions at zero temperature always exist for any 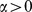 and 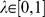. For non-zero temperature, the first order series expansion of Eqs. (20) with 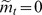 gives
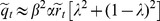


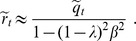



From the above equations it follows that the spin-glass transition temperature 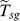 is
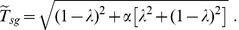
(22)


**Figure 12:**
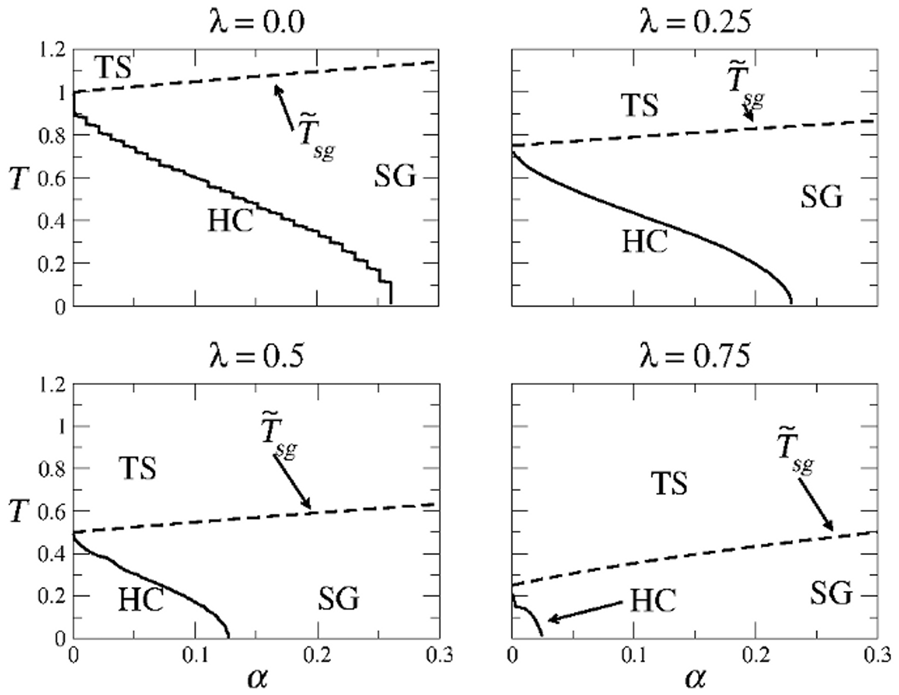
Structure of the SPR phase space for different values of 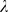
 **in the modified CS model.** The first panel corresponds to the well known asymmetric Hopfield model. Note the remarkable symmetry with respect to Fig. 11.Detailed calculations of the path integral method for the AM solutions with one set of patterns.

Figure 12 summarizes the structure of the 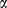-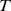 space in the SPR case for different values of 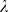. The usual three regions HC (high correlations), SG (spin-glass solutions only), and TS (trivial solution) are indicated. The boundary between regions SG and TS is given by Eq. (22), whereas the curve separating regions HC and SG was obtained numerically from Eqs. (20). Note that region HC remains of considerable size for a wide range of values of 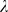.

## Discussion

We have obtained the complete phase diagram and analyzed the transitions from AM to SPR in a neural network model proposed by Coolen and Sherrington in which both types of pattern retrieval compete, as well as in a simple modification of the model in which both types of pattern retrieval may coexist. In these systems, the AM and SPR dynamics are encoded in the symmetric and asymmetric parts of the synaptic matrix, respectively, and the contribution of each of these parts is weighted by a parameter 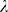 in such a way that when 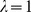 only the symmetric part survives, whereas when 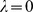 only the asymmetric part is present. In the original Coolen and Sherrington model, the same set of patterns are used to define the symmetric and asymmetric parts of the synaptic matrix. Using the standard functional generating formalism, we obtained the phase diagram of the system which shows that in the original Coolen-Sherrington model AM and SPR dynamics cannot coexist. This is simply due to the fact that a given pattern 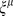 cannot be a fixed point and part of a larger cyclic attractor at the same time. Therefore, the original CS model can retrieve patterns only for the limiting cases 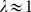 or 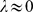. For intermediate values of 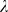 the network is “frustrated” and can perform neither AM nor SPR.

To prevent the system from falling into a “frustrated” state as mentioned above, we modified the CS model by using two independent sets of patterns in order to define separately the symmetric and asymmetric parts of the synaptic matrix. In doing so we allow the possibility for the network to have fixed points belonging to one set of patterns, and simultaneously cyclic attractors constructed with the patterns that belong to the other set. Our goal was to determine how the network transits from AM to SPR as 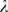 varies from 1 to 0 in this new case where the two sets of patters were independent. As expected, in this case the AM and SPR dynamics coexist for a wide range of values of 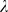. However, some other aspects of the model can be analyzed. For instance, quasi periodic states are known to occur in the original CS model (with only one set of patterns) and it would be interesting to determine to what extent these quasi periodic states exist in the modified CS model (with two independent sets of patterns). Also, it is possible to have an intermediate situation in which the two sets 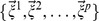 and 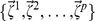 share some of the patterns. In this case the two sets would not be fully independent and the transition from AM to SPR dynamics could be more complicated.

Finally, the generating functional approach that we used to determine the structure of the phase space works very well when the patterns are uncorrelated and the network is fully connected. It would also be interesting to extend the analysis to networks with more realistic topologies, such as the small-world and scale-free topologies, in order to determine how the network topology affects the dynamics.

## Supporting Information

Appendix S1 (TEX)
